# Bi-objective location-allocation model of interventions in high drug consumption areas incorporating X topic modeling

**DOI:** 10.1007/s10729-025-09753-3

**Published:** 2026-05-08

**Authors:** Kevin Palomino, Carmen Berdugo, Jorge Acuña, José L. Zayas-Castro

**Affiliations:** 1 Department of Industrial Engineering, Universidad del Norte, Km 5 via Puerto Colombia, Barranquilla, Atlántico 100190 Colombia; 2 Faculty of Engineering and Sciences, Universidad Adolfo Ibáñez, Av. Padre Hurtado 750, Viña del Mar, Valparaíso 2510000 Chile; 3 Department of Industrial and Management Systems Engineering, University of South of Florida, 4202 E. Fowler Avenue, Tampa, Florida 610101 USA; 4 Department of Industrial Engineering, Universidad libre Barranquilla, Carrera 46 48-170, Barranquilla, Atlántico 080001 Colombia

**Keywords:** Drug abuse, Treatment centers, Multi-objective, Consumption risk, Location allocation

## Abstract

The Comprehensive Policy for the Prevention and Care of Psychoactive Substance Use in Colombia aims to improve the care provided to people, families, and communities at risk or struggling with psychoactive substance use through prevention and mitigation programs. The effectiveness of these programs depends on both population participation and access to intervention centers. This study proposes a bi-objective integer programming model within a location-allocation framework to support policy decisions under budget constraints. To estimate drug-related risk, we integrate sentiment analysis from social media data (X, formerly Twitter) as a key input into the optimization model. Specifically, negative sentiment derived from posts is used to inform the spatial distribution of risk between locations. The model simultaneously minimizes population-level risk and distance to services, while ensuring equitable coverage based on multidimensional poverty and rurality. The proposed approach was applied to real-world data from Atlántico. The results demonstrated that the bi-objective model achieved an average coverage of 24.67% of the population within a 40 km radius, effectively balancing service accessibility between high-risk urban areas and underserved rural zones. Compared to a population-based heuristic, which achieved only 8.85% coverage and excluded 22 of 23 locations, the proposed model significantly improved equity in service distribution. Furthermore, complementary topic modeling using Latent Dirichlet Allocation (LDA) revealed key themes in public discourse, including drug cartels, addiction risks, and social impacts, providing valuable information to support tailored communication and community engagement strategies for new intervention centers.

**Supplementary Information:**

The online version contains supplementary material available at 10.1007/s10729-025-09753-3.

## Highlights

A bi-objective location-allocation model was developed to optimize drug abuse prevention and mitigation services.

The model balances risk reduction and social equity, improving service coverage nearly threefold compared to population-based heuristics.

Social media sentiment analysis was integrated as a complementary risk indicator to inform resource allocation decisions.

Sensitivity analyses confirmed the robustness of the model under varying risk-equity priorities and budget scenarios.

The topic modeling of over 4,000 public posts revealed community concerns, supporting targeted, context-sensitive intervention strategies.

**Supplementary Information:**

The online version contains supplementary material available at 10.1007/s10729-025-09753-3.

## Introduction

Drug overdose deaths represent a global public health challenge, ranking as the leading cause of unintentional mortality worldwide. Some epidemiological data reveal that approximately 61 million people participated in drug use in 2020, underscoring the scale of this pervasive challenge [1, 2]. Contrary to common perception, this epidemic does not concentrate solely in metropolitan cities but has demonstrated particularly alarming trajectories in rural areas [3]. Each year, illicit drugs contribute to an estimated 11.8 million deaths globally [4]. In the United States, overdose-related deaths exceeded 100,000 in 2021, of which more than 75,000 were opioid overdoses, as reported by the Centers for Disease Control and Prevention [5]. Colombia also faces this challenge, recording approximately 28,541 deaths from psychoactive substance use between 2013 and 2020, indicating an average of about 10 daily deaths related to such consumption [6]. Furthermore, about $68\%$ of these drug overdose deaths can be attributed to opioid use, highlighting the severity of the opioid crisis [7, 8].

Drug addiction is a complex and chronic condition that requires an integrated response that addresses its physiological, psychological, and social dimensions. A variety of treatment modalities have been implemented to support individuals affected by drug use. Pharmacological interventions involve the use of medications to manage drug addiction [9]; behavioral therapies focus on identifying and modifying negative beliefs and actions related to drug use [10]; residential treatment programs provide a structured and supportive environment for people to receive intensive treatment for drug addiction [11]; peer support and mutual aid groups offer a supportive community, opportunities to share experiences and guidance to maintain abstinence [12]. Integrated approaches combining pharmacological, behavioral, and psychosocial strategies have shown particular effectiveness in addressing the multifaceted needs of individuals with substance use disorders [13].

Recent years have also seen the emergence of machine learning and geospatial analytics as valuable tools in identifying high-risk populations and improving decision making in drug prevention and treatment programs [14–16]. The use of early warning systems, informed by social media data and geolocated trends, has helped reveal spatial and temporal patterns of drug-related behavior [17–19]. Other implementations, such as community-based interventions, have gained prominence for their effectiveness in engaging local communities in addressing these issues [20–22]. Additional initiatives such as increased access to treatment services, targeted awareness campaigns, and comprehensive harm reduction programs have contributed significantly to reducing drug consumption and related harms [23, 25, 26]. Complementing these efforts, changes in policy and international collaborative efforts have collectively made progress in the prevention and mitigation of drug consumption [27–29]. However, despite these advances, the issue of drug consumption continues to demand sustained attention and innovative solutions from public health authorities and the international community.

Although there are various treatment approaches for drug addiction, their ability to address the multiple psychological, physiological, and social aspects of the condition depends on the accessibility and availability of suitable treatment facilities. Several research efforts have focused on reducing the distance between patients and care facilities [30, 31]. Others, considering congestion, simultaneously determine the location and size of facilities while allocating patients to preventive healthcare units [32–34]. Additional work has emphasized the importance of balancing equity and efficiency, incorporating measures such as minimizing travel distance and targeting underserved communities [37, 38]. Research in this area has also focused on addressing the healthcare facility location problem to maximize demand satisfaction through the quality of the center’s service[39, 40]. Several articles also consider variables such as total hospital charges, length of stay, and discharge destination to plan the location of health centers [41–43].

While considerable research has been dedicated to optimizing the location of healthcare facilities, much of this work focuses on general health services. In contrast, location-allocation models that specifically target drug-related facilities for prevention, treatment, or harm reduction have received comparatively limited attention. However, recent studies have begun to address this critical gap, applying operations research and optimization tools to improve the locations of the intervention in the context of substance abuse. For example, one study optimized treatment facility locations for substance abuse services in Oklahoma, evaluating the influence of different distance metrics such as Haversine, Euclidean, and Manhattan [44]. Another proposed an optimization model to improve the return and secure disposal of prescription opioids, in order to reduce their diversion to secondary users and the black market [45]. Additional research introduced an equitable, data-driven approach to resource allocation for opioid response efforts, focusing on balancing fairness and accessibility [46]. Finally, an optimization framework was developed for rapid overdose response using a drone delivery network [47].

### Summary of gaps

Despite the extensive coverage of geographical location and the reduction in distances between healthcare centers and patients in the academic literature discussed above [14–23, 25–34, 37–43], there is still a significant gap. Existing models often ignore social network interactions, including drug consumption, which are crucial for understanding community perspectives and opinions about drugs. Furthermore, the literature lacks a comprehensive model that simultaneously optimizes healthcare center locations based on distance minimization and risk factors, and integrates sentiment analysis to extract insights from consumer thoughts and opinions. As highlighted by [48], incorporating consumer perspectives into healthcare service optimization is essential to gain valuable information on the preferences, needs, and experiences of individuals seeking treatment for drug abuse. Understanding consumer perspectives can offer detailed insights into factors like accessibility, service acceptability, cultural considerations, and treatment preferences, all of which are crucial for designing patient-centered interventions [49].

### Research goals

The summary above establishes the need for an evidence-based approach that provides decision makers with a solid foundation for the location-allocation of centers, equitable geographical distribution and reduces the distance between patients and facilities while creating a valuable resource for decision makers and health officials to design more effective prevention and mitigation strategies. This work contributes to the literature by designing a bi-objective integer programming model that integrates a sentiment analysis to improve access to interventions, particularly in geographical areas with higher drug consumption, and extracts valuable information from social media to assist in designing more effective prevention and mitigation treatments. Our case study focuses on Colombia; however, the mathematical framework and methodologies can be contextually adapted and adopted to countries with similar structures. Concretely, the study aims to:

- Develop a framework to determine the optimal location of the intervention centers, taking into account both risk and equity simultaneously.
- Generate a balanced allocation of government resources that minimizes travel distances for both prevention and mitigation interventions.
- Implement topic modeling to extract valuable insights from social media data, capturing the perspectives and opinions of people who use drugs.

### Relevance

This research holds significant relevance for policymakers and public health officials, providing them with a valuable resource for designing more effective prevention and mitigation treatments targeted at communities with drug addiction. Using insights extracted from social network interactions, our model offers an understanding of the perspective of the drug community. Ultimately, this research contributes to improving the health outcomes of people affected by drug addiction, addressing a pressing public health problem. Moreover, the incorporation of both geographical distribution and topical relevance in our model ensures its applicability and effectiveness in real-world scenarios, making it relevant for planners, hospital managers, and regulatory bodies tasked with addressing drug-related challenges.

## Model formulation

The network design problem determines the location and capacity of facilities that serve population zones with the demand for their services [31]. The need for an efficient system with high quality of service and the effort to maintain a balanced spatial distribution of services, even in the most remote areas (equity), make the location-allocation of healthcare facilities multi-objective in nature [32]. Unfortunately, the unequal spatial distribution of facilities creates a significant challenge, limiting access for people who reside far from the centers due to increased travel costs and times. To address this challenge and achieve optimal network design, we focus on two key factors: efficiency and equity in facility location. An effective system maximizes social welfare (minimizes risk) in high-consumption areas under fixed budgets. The principle of equity imposes that access costs to equal welfare opportunities should be evenly distributed [33]. Therefore, the optimal health provision system must be spatially fair and efficient. To preserve the transparency of trade-offs and support decision-making across diverse stakeholder priorities, we modeled risk and equity as separate objectives rather than combining them into a single composite function. This formulation allows exploration of the Pareto frontier, enabling planners to visualize the implications of prioritizing one goal over another.

The model’s assumptions are: i) health centers are capacitated; ii) the time and distance between the demand node and the health center nodes are constant; and iii) serving more patients decreases the consumption risk. The proposed bi-objective integer programming (BI-IP) location-allocation model is formulated as follows:

The first objective function (OF1) aims to maximize the weighted sum ($\delta$) of two factors: the total patient welfare and the normalized negative distance ($\frac{-d_{i,j}}{D_{Max}}$) for patient flow ($Y^k_{ij}$) between the demand node *i* and the center node *j* (Equation 1). Here, ($d_{i,j}$) represents the distance between the demand node *i* and the center node *j*, indicating the shortest route for patient flow. Additionally, $Y^k_{ij}$ is an integer variable that represents the number of patients assigned from demand node *i* to center node *j*; in other words, it quantifies the flow of patients between these nodes. The weight parameter $\delta$ adjusts the importance of optimizing patient welfare relative to minimizing distance, thus balancing the objectives of maximizing welfare and minimizing travel distance within the optimization model. To facilitate reading, Tables 1 and 2 summarize the parameters and decision variables, respectively.

1 $$\begin{aligned} \text {Max OF1} =\delta (\sum _{i \in I}\sum _{j \in J}\sum _{k \in K} R^k_{i}*Y^k_{ij})+ (1-\delta )\sum _{i \in I}\sum _{j \in J}\sum _{k \in K}[\frac{-d_{ij}}{D_{Max}}]*Y^k_{ij} \end{aligned}$$

The total welfare of the patient is defined as the number of individuals served at the demand node *i*, weighed by their relative risk of psychoactive substance use. The risk index ($R^k_{i}$) proposed in this research is calculated as the average percent demand for the psychoactive substance (${\alpha _i}$), the percentage of negative posts on social networks related to drug consumption (${\psi _i}$), and the risk factors (${\gamma _i}$) in the node *i* (Equation 2). The risk factor (${\gamma _i}$) can be understood as including any individual, social, or environmental factors (such as violence, crime, robbery, etc.) that increase the likelihood of drug use [34]. The inclusion of social media sentiment ($\psi _i$) is supported by studies showing that expressions on platforms like X correlate with behavioral health risks. For example, a scoping review by [35] discusses the value of social media analysis for the detection and pharmacovigilance of adverse events, highlighting the potential of social media data in monitoring public health concerns. Although this study focuses on pharmacovigilance, the methodologies, and insights are applicable to analyzing public sentiment related to drug use.

Interpreting higher levels of negative sentiment as indicative of increased patient risk is based on the understanding that expressions of fear, frustration, or concern about drug-related issues on social networks often reflect elevated community distress or perceived vulnerability. In public health research, such sentiment signals are commonly used to identify areas with greater unmet needs or psychosocial stressors. Therefore, in this study, it is assumed that locations with a higher proportion of negative sentiment posts experience heightened risk environments, and this information is incorporated into the risk index ($\psi _i$) accordingly.

To estimate the risk index associated with prevention, we adopt the assumption that for every two individuals at mitigation risk, there is one person at prevention risk, expressed as $R_i^2 = \frac{1}{2}R_i^1$, where $k = 1$ (representing prevention) and $k = 2$ (representing mitigation). In this context, prevention seeks to prevent people from starting to use drugs, and mitigation focuses on reducing the harms associated with drug use for people who are already using.

2 $$\begin{aligned} R_i^{k=1} =\frac{{\alpha _i}+{\psi _i}+{\gamma _i}}{3} \end{aligned}$$

The formulation of the risk index ($R^k_i$) using an unweighted arithmetic mean follows established practices in the construction of composite indices. This approach assumes that each component (substance demand, social sentiment, and contextual risk) contributes equally to the overall level of risk, ensuring transparency and avoiding the introduction of subjective bias. This method is commonly applied when there is no strong empirical or theoretical justification to prioritize one component over another. It provides a balanced, easily interpretable measure that facilitates cross-comparison and aligns with approaches used in public health, quality of life studies, and social vulnerability assessments. For example, [36] highlights the utility of composite indices with unweighted structures for summarizing health data in population studies.

The distance is defined as the shortest driving distance from the demand node *i* to the health center *j*. An algorithm was designed that calculates the distance between nodes using the GeoJSON API of the web mapping service developed by Google Maps, derived from [37]. This algorithm was based on the possibility of accessing public cartography and accurate road network data for specific study problems and then exporting these data to a database. Similarly, the second objective function (OF2) maximizes the weighted ($\pi$) sum of equity and the normalized negative distance (Equation 3). Although objective functions are often expressed in minimization form, we adopt a maximization approach to emphasize gains in coverage, risk mitigation, and equity in resource distribution. Since maximizing a positive impact (e.g., patients reached or risk addressed) is mathematically equivalent to minimizing a cost or loss, the formulation remains valid and interpretable.

3 $$\begin{aligned} \text {Max OF2} =\pi (\sum _{i \in I}\sum _{j \in J}\sum _{k \in K} \sigma ^k_{i}*Y^k_{ij})+ (1-\pi )\sum _{i \in I}\sum _{j \in J}\sum _{k \in K}[\frac{-d_{ij}}{Max\ (d_{ij})}]*Y^k_{ij} \end{aligned}$$

The equity index ($\sigma ^k_{i}$) proposed in this research corresponds to the multiplication of the multidimensional poverty index (MPI) and the rurality proportion (RP) in node *i* (Equation 4). This multiplicative formulation is intentional and reflects the compounded effect of socioeconomic deprivation and geographic isolation. Unlike an additive approach, multiplication ensures that areas with high poverty and high rurality are emphasized, aligning with equity-driven planning goals.

Multiplicative aggregation is a widely recognized method in the development of composite indices, particularly in the context of poverty measurement. It offers an advantage by avoiding arbitrarily weighting and reducing substitutability between indicators. This approach ensures that a high value in one indicator cannot compensate for a low value in another, thereby providing a more rigorous and precise assessment of multidimensional disadvantages. This structure aligns with the Multidimensional Poverty Index, which multiplies the incidence and intensity of deprivation. Similar methods are discussed in the literature on poverty and health equity, where composite indices are used to target underserved populations [24].

To differentiate between prevention and mitigation priorities, we adopt the assumption $\sigma ^2_{i}=\frac{1}{2}\sigma ^1_{i}$, where $k = 1$ represents prevention and $k = 2$ mitigation.

4 $$\begin{aligned} \sigma _i^{k=1}=RP_i*MPI_i \end{aligned}$$

Although both objective functions include a distance term, they represent distinct planning goals. Objective Function 1 considers distance as part of a composite score alongside risk to prioritize interventions in high-need areas, while Objective Function 2 uses distance to reduce spatial inequality in access. This design ensures that all optimized solutions account for proximity, regardless of which objective is prioritized.

A demand constraint was required to ensure that all flows from node *i* to the healthcare center *j* align closely with the specified demand $D_i^k$ (see Equation 5). The MPI provides an equitable representation by capturing the complex realities of impoverished individuals and their communities, addressing the individual and collective aspects of poverty [38]. Generally, the poorest live in areas with difficult access (non-urban) and relatively low living conditions [50].

5 $$\begin{aligned} \sum _{j \in J} Y^k_{ij} \le D^k_i, \ \ \ \ \ \ \ \ \forall i \in I , k \in K \end{aligned}$$

In the same way, Equation 6 is the budget constraint. It ensures that all financial resources do not exceed the funds allocated to prevent and mitigate the consumption of psychoactive substances. Where $F^k$ represents the operational cost per hour of the center and $C^k_{j}$ the maximum number of patients that can be served per hour of capacity in the center *j*, related to the intervention *k*.

6 $$\begin{aligned} F^k\sum _{j \in J} C^k_{j} \le \text {Budget}^k, \ \ \ \ \ \ \ \ \ \ \ \ \forall k \in K \end{aligned}$$

Similarly, the model requires two additional constraints that define the capacity of each facility. The first is to determine the minimum working hours ($R_{min}$) to feasibly open a health center in node *j* (Equation 7), where $X_j$ is a binary variable, 1 if the center is open, 0 otherwise.

7 $$\begin{aligned} \sum _{k \in K} C^k_{j} \ge R_{min}*X_j, \ \ \ \ \ \ \ \ \ \ \ \ \forall j \in J \end{aligned}$$

The second constraint (Equation 8) ensures that the total service time required to attend to all assigned patients does not exceed the available capacity in each health center. Where $L^k$ is the number of hours required per patient of the intervention type *k*.

8 $$\begin{aligned} L^k\sum _{i \in I} Y^k_{ij} \le C^k_{j}, \ \ \ \ \ \ \ \ \ \ \ \ \forall j \in J, k \in K \end{aligned}$$

Furthermore, it was considered essential that the capacity of a center is only calculated when the center is open. In Equation 9, *M* is a big number. In selecting an optimal value for *M*, it is advisable to refrain from setting it excessively high, as this may lead to undesirable delays in the solution process. A more practical approach involves defining *M* as the sum of the total demand, as it ensures that the flow through each arc never exceeds the cumulative demand requirement.

9 $$\begin{aligned} \sum _{i \in I} \sum _{k \in K} Y^k_{ij} \le M*X_j, \ \ \ \ \ \ \ \ \ \ \ \ \forall j \in J \end{aligned}$$

Finally, equity constraints are considered (Equation 10). Based on the assumption derived from the public mental health policy, the benefits should be fairly distributed among the population [51]. At least $\varphi$ patients should be attended to at each demand node *i*. This constraint ensures that the model cannot assign zero patient flow to any demand node, even in cases where distance is heavily weighted in the objective function.

10 $$\begin{aligned} \sum _{j \in J} \sum _{k \in K} Y^k_{ij} \ge \varphi , \ \ \ \ \ \ \ \ \ \ \ \ \forall i \in I \end{aligned}$$

**Table 1:** Sets and parameters in the model

Notation	Definition
*I*	Set for demand node indexed by *i*
*J*	Set of candidate locations to build new health centers, indexed by *j*
*k*	Set for intervention type indexed by *k*
$R^k_i$	Risk of the node *i* related to intervention *k*
$\sigma ^k_{i}$	Equity index of the node *i* related to intervention *k*
$d_{ij}$	Distance between demand node *i* and center *j*
$D^k_{i}$	Demand of the area *i* related to intervention *k*
$\text {Budget}^k$	Maximum funding available for intervention *k*
$F^k$	Hourly cost of center *j* related to intervention *k*
$\delta$	Weights for the risk and distance in the objective function 1; $\delta _1 + \delta _2=1$
$\gamma _i$	Proportion of risk factors in the area *i* related to psychoactive substance consumption
$\alpha _i$	Proportion of psychoactive substance consumers in the area *i*
$\pi$	Weights for the equity and distance in the objective function 2; $\pi _1 + \pi _2=1$
$\psi _i$	Proportion of negative post in the area *i* related to psychoactive substance consumption
$\varphi$	Minimum number of patients to be served at the demand node *i*
$L^k$	Number of hours required per patient related to intervention *k*.
$R_{min}$	Minimum number of working hours in a center
*M*	Big number

**Table 2:** Decision variables in the model

Notation	Definition
$Y^k_{ij}$	Number of patients assigned from the demand node *i* to center node *j*
$C^k_{j}$	Hourly capacity of the center *j* for intervention *k*
$X_j$	Binary variable equal to 1, if a candidate location *j* is opened; 0, otherwise

To solve the proposed bi-objective model, we applied the epsilon constraint method, a standard approach for generating Pareto optimal solutions in multi-objective optimization problems. For additional examples of multi-objective optimization models applied in the healthcare domain, see [52, 53]. This method reformulates the original problem into a series of single-objective optimizations by constraining one objective while optimizing the other. Each run generates a point on the Pareto frontier, allowing for the identification of optimal trade-offs between conflicting objectives, such as risk and equity, in our model. The mathematical formulation and detailed pseudocode of the algorithm are provided in Appendix A.

## Case study

The study was conducted in Atlántico, Colombia, a department (i.e., province or state) located in the northern part of the country within the Caribbean region. Geographically, it is between 10°15’36" and 11°06’37" north latitude and 74°42’47" and 75°16’34" west longitude. Covering an area of 3,386 $km^2$, Atlántico represents 0.29% of the national territory. The state is composed of 22 municipalities, with Barranquilla being its capital. Approximately 2.7 million people live in Atlántico (5.4% of the total population of the country), with 94.8% residing in urban areas, while the remaining 5.2% inhabit rural regions [54].

**Fig. 1:**
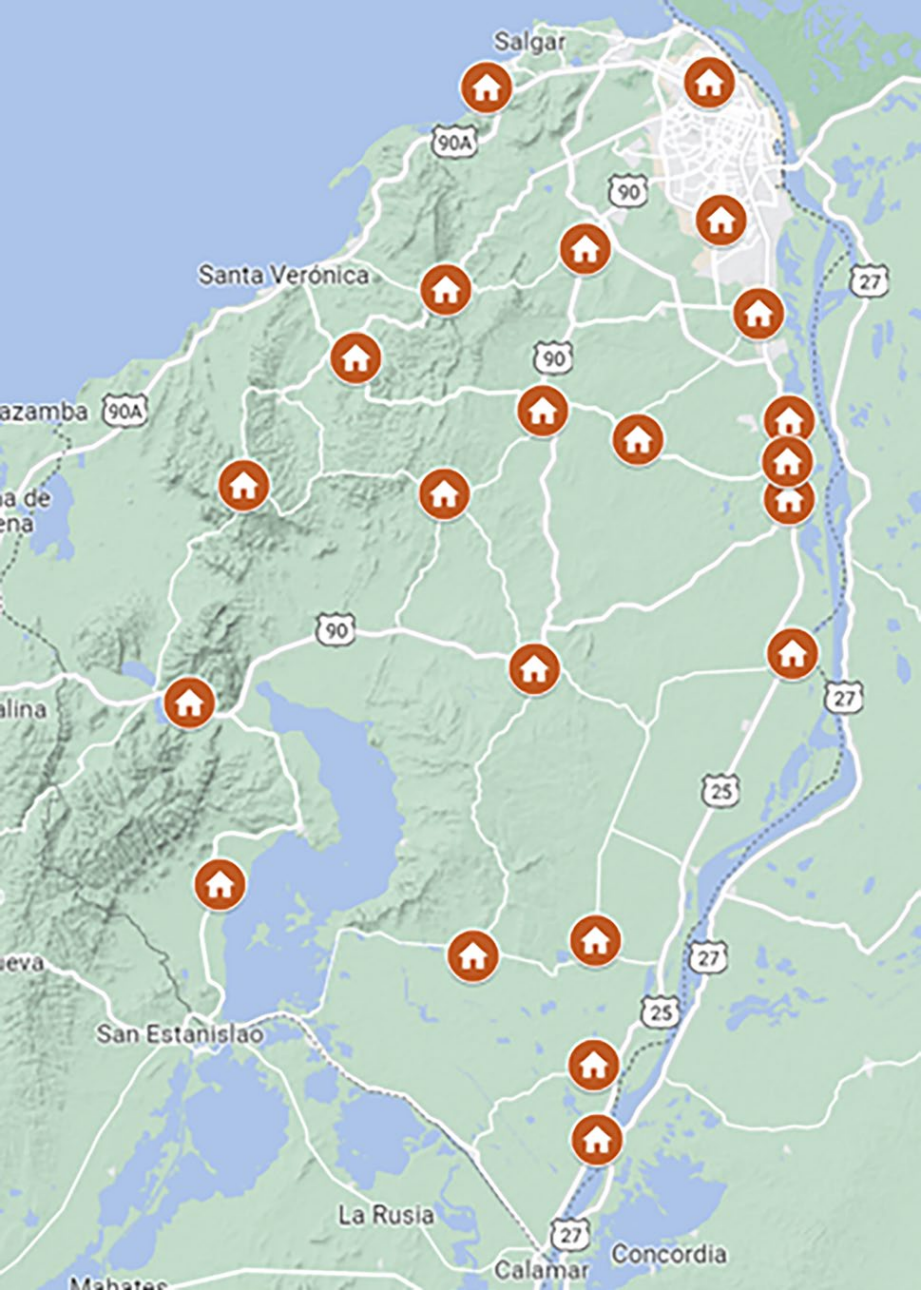
Potential locations for health centers

In terms of economic development, Atlántico is a key player in the Caribbean region, boasting a gross domestic product (GDP) that exceeds the national average [55]. However, despite this economic prosperity, there is considerable consumption of non-prescription tranquilizers, opioids, ketamine, GHB, alcohol, and heroin [16, 56]. Given that opioids are the most prevalent drug in Atlántico [16], this research focuses only on applying the model to address issues related to opioids (constituting 52% of total drug consumption). To model the area’s demand, the study computed the centroid of each municipality, represented by a house icon (see Fig. 1). These centroid locations serve as potential candidates for the establishment of health centers within the model.

### Model parameters

 

**Fig. 2:**
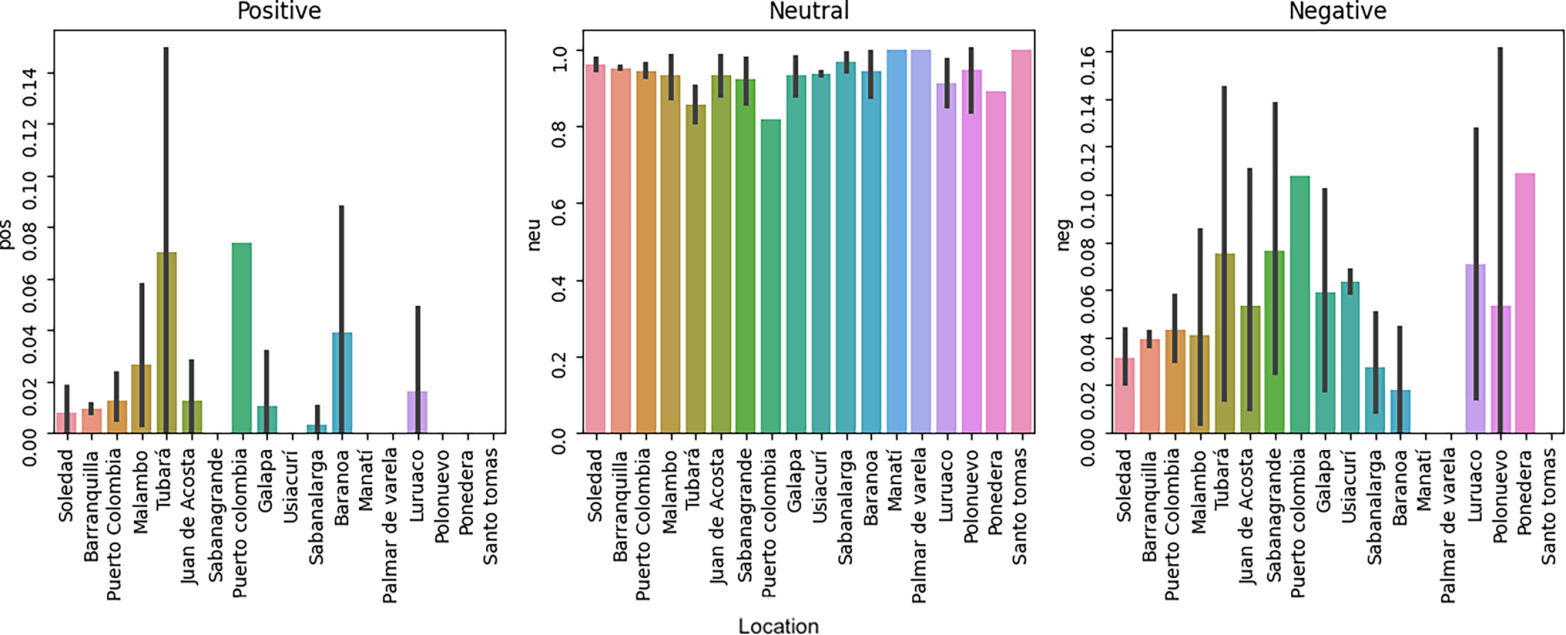
Distribution of positive, neutral, and negative comments per location

**Fig. 3:**
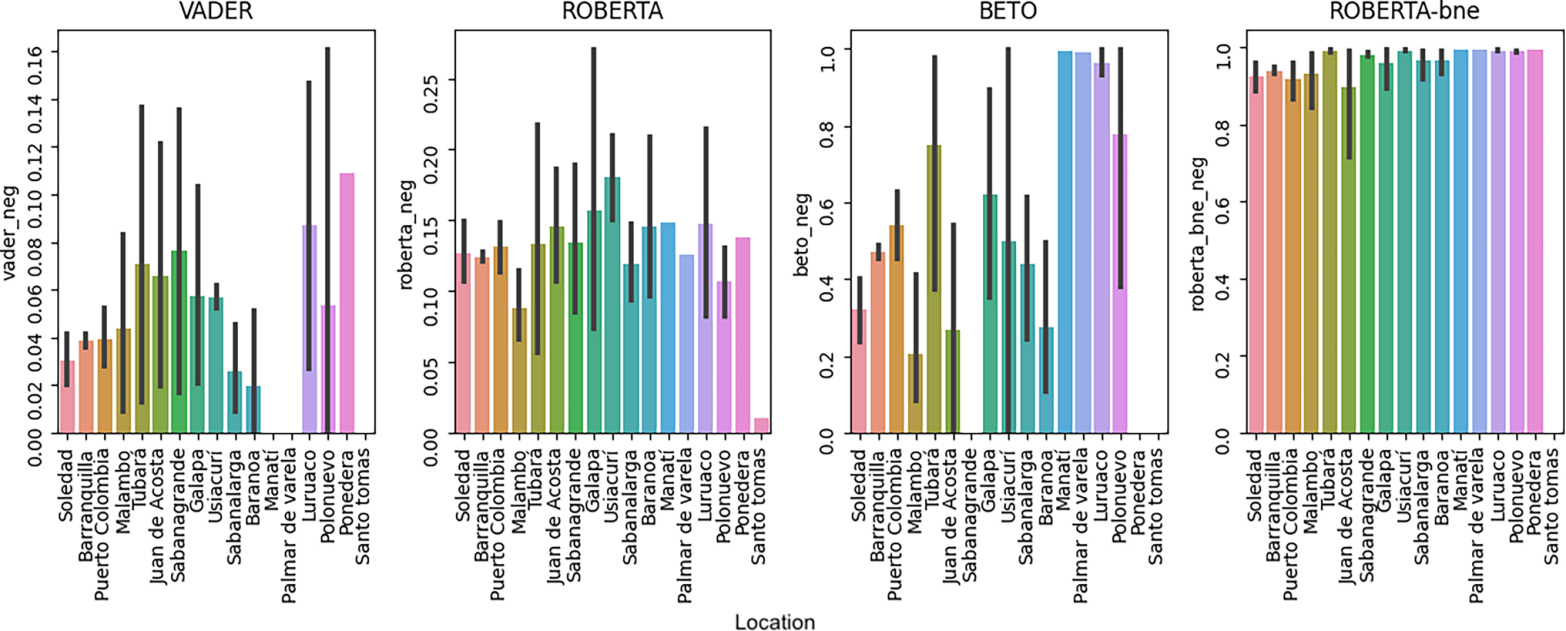
Sentiment algorithms results

- Demand ($D_i^k$): We used information retrieved from the 2019 National Survey on Psychoactive Substance Consumption in the General Population (DANE-DIMPE-ENCSPA-2019) conducted by the Colombian National Statistical System (DANE) to estimate the consumption demand of each location [54, 56]. In this regard, the consumption demand was calculated as the count of people using drugs in each city in Atlántico during 2019. We consider at-risk people who are older than 15 years old. According to DANE, 68.26% of the people in Colombia are between 15 and 64 years old.
- Psychoactive substance risk ($\alpha _i$): This parameter reflects the relative intensity of drug use in municipalities. It was calculated by first estimating the total number of individuals consuming psychoactive substances in each municipality [56]. The risk value $\alpha _i$ was then calculated as the number of estimated consumptions of each municipality divided by the total estimated consumption in all municipalities.
- Distance ($d_{ij}$): Computing the distance between supply and demand nodes is essential for the model, which requires network structures and routing algorithms [57]. However, data availability can be challenging. To address this, a Python code integrated with the GeoJSON API was developed. This code collects real-distance data from the road network for any case study and records it in a database. Google’s API distance matrix provides distance and travel time for a matrix of origins and destinations [57]. By default, distances are calculated for the driving mode using the road network. In addition, distance values may be subject to certain restrictions (tolls, highways, ferries, indoor, or default: null). No restrictions were included in this code. The units (metric) for the distance display are determined by the query’s origin country.
- Budget ($B^k$): We used the information that the Colombian Ministry of Health presented to determine the available funds to reduce the use of the psychoactive substance [58]. Then, to estimate the total budget to prevent and mitigate the consumption of psychoactive substances, we used the distribution of financial resources by state [59]. In total, 3 billion Colombian pesos (COP) were allocated for mitigation campaigns and 654 million for prevention.
- Multidimensional poverty index ($MPI_i$): The MPI values for each state were retrieved from the multidimensional poverty report presented by the National Statistical System (DANE) [60].
- Cost of service per hour ($F^k$): The monthly pay for a psychiatrist in Colombia is COP 6.1 million, while the average salary of a psychologist is COP 1.8 million [61]. The role of a psychiatrist involves administering mitigation treatments, while a psychologist focuses on providing prevention treatments. It is assumed that the professionals work for eight hours a day, twenty days a month.
- Weights in the objective functions ($\pi ,\delta$): The purpose of using weights is to balance the different component of each objective functions. In the case of objective function 1, it considers the number of people served at risk, and the total distance traveled, with $\delta$ being the controlling parameter. The research uses a value of $\delta = 0.7$. This value was chosen to reflect a deliberate prioritization of social welfare considerations, but this value can vary according to how important the distance or the risk is relative to each other. Although this choice is arbitrary, it aligns with the objective of the study to support decision-making in vulnerable populations. Similarly, objective function two is treated analogously, with the parameter $\pi$ being used.
- Minimum number of working hours in a center ($R_{min}$): This value represents the number of hours per year in minimum capacity. Thus, it is estimated based on a shift of eight hours per day for 260 days per year. A distribution of equal hours for mitigation and prevention is assumed.
- Number of persons per hour of therapy ($L^k$): Based on interviews with two healthcare professionals from organizations that specialize in psychoactive substance treatment, mitigation therapy is administered individually and typically lasts one hour per patient. In contrast, prevention therapy is also one hour long but is conducted in group sessions serving 20 individuals. To estimate facility capacity, we assumed one full-time professional per center working 8 hours per day for 260 days per year, totaling 2,080 annual service hours. Although these service times ($L^k$) are treated as fixed inputs, the number of professionals required per facility is not predetermined; rather, it is computed endogenously by the optimization model according to demand and capacity constraints.
- Risk factor ($\gamma _i$): A risk factor refers to any variable or characteristic that increases the likelihood that an individual develops a substance use disorder or experiences negative consequences due to substance use. In this study, we estimate this value based on the proportion of homicides, thefts, terrorism, sexual crimes, domestic violence, and threats in each municipality. This information was retrieved from the databases of the Colombian National Police [62].
- Negative post-risk on social networks ($\psi _i$): Refers to the potential harm or influence that posts containing negative sentiment (such as anger, fear, or criticism) can have on individuals or communities. Based on the data extraction method proposed in Appendix B, we first identify the sentiment polarity of posts based on their geolocation using four pretrained sentiment analysis models: Robustly Optimized BERT Pretraining Approach (Roberta) [63, 64]; Valence Aware Dictionary for Sentiment Reasoner (VADER) [65]; Bidirectional Encoder Representations from Transformers model trained on a Spanish corpus (BETO) [66]; and Roberta-base-bne [67]. Figure 2 displays the distribution of positive, neutral, and negative posts by location, while Fig. 3 shows the average probability of negative classification across the four algorithms. In particular, RoBERTa-base-bne consistently assigned higher probabilities for negative sentiment. However, it is important to interpret these figures in conjunction with Table 3, which reports the number of posts per location. For example, Ponedera appears to have a high proportion of negative posts in Fig. 3, but this may be misleading as it is based on only one post. The final risk score $\psi _i$ is calculated as the proportion of negative posts in each location relative to the total number of negative posts in all locations.

**Table 3:** Distribution of posts per location

Municipality	posts
Barranquilla	2741
Baranoa	18
Campo de la cruz	-
Candelaria	-
Galapa	11
Juan de acosta	11
Luruaco	-
Malambo	24
Manatí	1
Palmar de varela	1
Piojó	-
Polonuevo	5
Ponedera	1
Puerto Colombia	108
Repelón	-
Sabangrande	8
Sabanalarga	24
Santa Lucía	-
Santo Tomás	1
Soledad	124
Suan	-
Tubará	5
Usiacurí	2

### Computational results and insights

This section presents the results and illustrates the complex trade-offs between fairness (equity) and coverage (risk) that emerge when designing a resource-constrained healthcare network. The computational experiments were performed using Python V.3.10 and Gurobi Optimizer V 9.5.2. The proposed algorithm converged to a solution with an optimality gap close to $10^{-4}$.

**Fig. 4:**
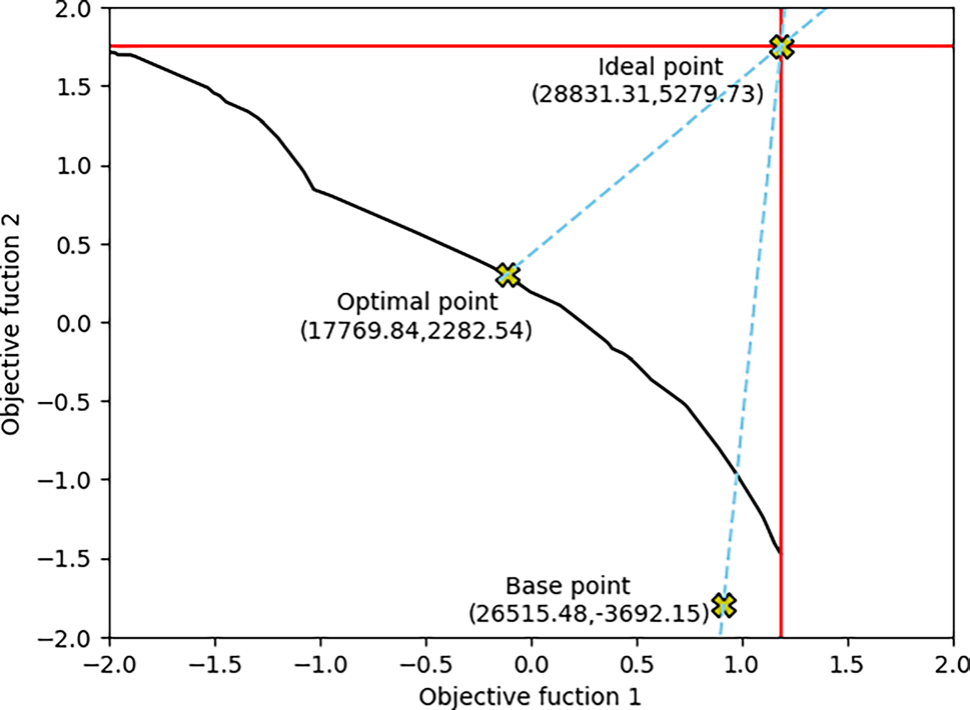
Pareto frontier

As a result, the integer programming model presented in Section 4.3 has 1127 integers and 23 binary variables. The model was solved to exact optimality on a Core I5 machine with a 2.9 GHz processor and 16 GB RAM in 0.01s computational time for each run. Adopting the epsilon constraint method, we optimize the first objective while constraining the secondary objective within a range of feasible values. This process was repeated iteratively to generate the Pareto frontier. From the non-dominated frontier, the best trade-off solution was selected by calculating the minimum Euclidean distance to the ideal point, as described by [68, 69]. To account for the different units and value ranges between the objectives, we applied z-score normalization, where each value is transformed by subtracting the mean and dividing by the standard deviation of its corresponding objective. This standardization allows both objectives to be expressed on a comparable scale, facilitating an unbiased calculation of Euclidean distances. The trade-off between risk and equity produces efficient solutions, as shown in Fig. 4. For these solutions, it is not possible to improve one objective without deteriorating the other. The ideal point (28831.31, 5279.73) corresponds to solving each objective function independently; meanwhile, the optimal point (17769.84, 2282.54) corresponds to the point with the minimal distance between the ideal point and the Pareto frontier solution.

To compare the results of the proposed model, we implemented a heuristic approach that selects the service center locations based on the population size. In this heuristic, cities are prioritized in descending order of population under the assumption that larger populations indicate a higher demand for services. Service centers are assigned to the most populated cities until all available resources are fully allocated. In this sense, Barranquilla, the most populated city in Atlántico, is only selected as the location to open a center. Although this strategy may seem efficient from a population coverage perspective, it results in a highly unequal distribution of services. Only residents of Barranquilla benefit from proximity to care, while the remaining 22 locations are restricted from service access. For people in these underserved areas, travel times to Barranquilla can exceed an hour, depending on distance and transportation availability, creating substantial barriers to timely treatment and exacerbating existing health inequities. In contrast, the proposed location-allocation bi-objective model explicitly incorporates both drug consumption risk and social equity, promoting a more balanced and inclusive distribution of health services. The comparison highlights how the heuristic method can focus solely on population distribution, potentially overlooking areas of high drug risk or underserved rural areas, thus offering a valuable baseline for evaluating the effectiveness of the proposed model. The base point (26515.48, -3692.15) in Fig. 4 corresponds to the point generated using this population-based heuristic approach.

Table 4 shows the total number of service hours of each demand node to the health center for prevention. Considering only the first objective function, the solution indicates that two health centers (Barranquilla, Baranoa) must be established to serve people under preventive treatment. Meanwhile, the second objective function shows that the centers to be opened are Juan de Acosta and Sabanalarga. These independent solutions show a conflict between where to locate the intervention center and how to assign people to each center, the first trying to place the intervention in areas with high drug consumption and the second trying to set a center closer to rural zones. In this sense, the solution obtained from the bi-objective model presents a balance between the previous solutions, i.e., to consider setting up a health center in Barranquilla that assists zones with high drug risk and another center in Sabanalarga serving rural zones. The center in Barranquilla would serve Galapa, Malambo, Puerto Colombia, Soledad, Barranquilla, and Tubará, while the center in Sabanalarga would serve the remaining towns. In comparison, the population-based baseline model assigns both centers to the most populated city, Barranquilla. Consequently, Barranquilla receives 67,340 total service hours, while all other municipalities are limited to 2,000 hours each, the minimum required due to policy constraints. This allocation highlights how a purely population-driven approach, although efficient for high-density areas, severely limits service access for rural zones.

**Table 4:** Number of service hours for prevention strategy in each health center

	Bi-objective		Objective 1		Objective 2		Baseline
	Center 1:	Center 2:	Center 1:	Center 2:	Center 1:	Center 2:	Center:
	Barranquilla	Sabanalarga	Barranquilla	Baranoa	Juan de Acosta	Sabanalarga	Barranquilla
Barranquilla	29617	-	67340	-	2000	-	67340
Baranoa	-	2000	-	2000	2000	-	2000
Campo de La Cruz	-	2000	-	2000	-	2000	2000
Candelaria	-	2000	-	2000	-	2000	2000
Galapa	2000	-	-	2000	2000	-	2000
Juan de Acosta	-	2000	-	2000	14334	-	2000
Luruaco	-	2000	-	2000	-	19233	2000
Malambo	2000	-	-	2000	1993	7	2000
Manatí	-	2000	-	2000	-	2000	2000
Palmar de Varela	-	2000	-	2000	-	2000	2000
Piojó	-	2000	-	2000	4511	-	2000
Polonuevo	-	2000	-	2000	2000	-	2000
Ponedera	-	2000	-	2000		16279	2000
Puerto Colombia	2000	-	2000	-	2000	-	2000
Repelón	-	2000	-	2000	-	2000	2000
Sabanagrande	1983	17	-	2000	-	2000	2000
Sabanalarga	-	39723	-	2000	-	11121	2000
Santa Lucía	-	2000	-	2000	-	2000	2000
Santo Tomás	-	2000	-	2000	-	2000	2000
Soledad	2000	-	400	1600	2000	-	2000
Suan	-	2000	-	2000	-	2000	2000
Tubará	2000	-	-	2000	11862	-	2000
Usiacurí	-	2000	-	2000	-	2000	2000

Note: Prevention treatment serves 20 people, and each therapy takes one hour. The total number of service hours represents the annual distribution

Although both the bi-objective and Objective 1 solutions include a facility in Barranquilla, it is important to acknowledge the potential implementation risk that such a center may become overwhelmed by local demand. As the most populated city in the state, Barranquilla’s residents could occupy a disproportionate share of the capacity if the facility, potentially limiting access for populations from neighboring municipalities assigned to the same center by the model. This could create real-world mismatches between planned service allocation and actual usage. Future research should explore control mechanisms, such as referral policies or suballocations of facilities by municipality, to ensure that access remains balanced and equitable. This issue also highlights the need for close coordination with local health authorities to implement policy safeguards that preserve the equity goals of the bi-objective solution.

Concerning mitigation treatments, Table 5 shows the total number of service hours of each demand node to the health center. In this way, the center in Barranquilla would serve the population of Malambo, Barranquilla, Galapa, Sabanagrande, Puerto Colombia, Soledad, and Tubará. In contrast, the center in Sabanalarga would serve the remaining towns. It is essential to mention that this solution seeks to reduce travel distances from the demand node to each center and to reduce the risk of consumption in the target population. In contrast, the population-based baseline model results in a highly unequal distribution of mitigation services. Specifically, Barranquilla receives 20,137 service hours, while each of the remaining locations is allocated only 100 service hours, the minimum feasible under the model’s constraints.

**Table 5:** Number of service hours for mitigation strategy in each health center

	Bi-objective		Objective 1		Objective 2		Baseline
	Center 1:	Center 2:	Center 1:	Center 2:	Center 1:	Center 2:	Center:
	Barranquilla	Sabanalarga	Barranquilla	Baranoa	Juan de Acosta	Sabanalarga	Barranquilla
Barranquilla	11657	-	20137	-	100	-	20137
Baranoa	-	100	-	100	3057	-	100
Campo de La Cruz	-	100	-	100	-	100	100
Candelaria	-	784	-	100	-	784	100
Galapa	100	-	-	100	100	-	100
Juan de Acosta	-	100	-	100	1029	-	100
Luruaco	-	1381	-	100	-	1381	100
Malambo	100	-	-	100	100	-	100
Manatí	-	100	-	100	-	100	100
Palmar de Varela	-	100	-	100	-	100	100
Piojó	-	324	-	100	324	-	100
Polonuevo	-	100	-	100	892	-	100
Ponedera	-	1169	-	100	-	1169	100
Puerto Colombia	100	-	100	-	100	-	100
Repelón	-	100	-	100	-	1284	100
Sabanagrande	100	-	-	100	-	100	100
Sabanalarga	-	4570	-	100	-	4570	100
Santa Lucía	-	100	-	100	-	100	100
Santo Tomás	-	100	-	100	-	100	100
Soledad	100	-	20	80	100	-	100
Suan	-	100	-	100	-	100	100
Tubará	852	-	-	100	852	-	100
Usiacurí	-	100	-	100	-	100	100

On the other hand, Table 6 presents the percentage of demand covered within a 40 km range. In objective function 1, Piojó demonstrates coverage values of 44.34% and 30.86% for prevention and mitigation, respectively, while Barranquilla registers 8.18% and 34.07%. Suan follows with coverage of 24.54% and 17.06% for prevention and mitigation. These locations show the highest coverage percentages within a 40 km radius. With objective function 2, Piojó, Juan de Acosta, Luruaco, Ponedera, and Tubará achieve full coverage (100%) within the 40 km range. Consequently, the entire population served in these locations must travel no more than 40 km, equivalent to 40–50 minutes of public or private transportation. Regarding the baseline approach, only nearby areas like Polonuevo (16.10% prevention) and Barranquilla itself (34.06% mitigation) achieve moderate coverage. However, the rest of the locations (13 out of 23 ) remain significantly underserved.

It should be noted that in Table 6, some locations without a health center (e.g., Piojó, Tubará, Ponedera) achieve 100% coverage, while others with a center (e.g., Barranquilla, Sabanalarga) report coverage levels below 20% in the bi-objective model. Although this may seem counterintuitive, it reflects the population distribution in Atlántico. Locations that achieve full coverage typically have smaller, more concentrated populations, allowing them to be fully served by a nearby center without exceeding the capacity of the facility. However, large urban areas such as Barranquilla host a substantial share of the total population, making it impossible to achieve full coverage under current budget and capacity constraints. This highlights a vital planning consideration: equitable geographic placement of facilities does not necessarily equate to proportional population coverage, particularly when demand is heavily concentrated in specific areas. Therefore, decision makers must weigh the trade-offs between maximizing coverage percentages and ensuring that smaller, underserved municipalities also receive attention. This spatial disparity in coverage outcomes provides valuable context for interpreting the broader performance of the bi-objective model. Although some small locations benefit from full coverage due to their limited demand and proximity to service centers, larger urban areas remain only partially covered due to capacity and budget limitations.

Building on this, it becomes evident that even though specific locations achieve high local coverage, the overall average coverage across all areas under the bi-objective model remains relatively low. Specifically, the average percentage of the population covered within a 40 km radius is only 24.67%. This value reflects a compromise between two competing priorities, such as risk reduction and equity enhancement. It is calculated by averaging the percentage of the population within the range of a center for both prevention (14.06%) and mitigation (35.29%) services. The relatively low average can be attributed to several factors. First, budgetary constraints limit the number of health centers that can be established, only two in this case, resulting in large geographic areas that remain underserved. Second, the model aims to strike a balance between urban centers with high drug consumption (associated with higher absolute demand) and rural areas with higher multidimensional poverty and isolation (equity concerns). Because rural areas tend to have lower population densities, serving them can reduce the overall percentage of the population covered, even if it improves geographic and social fairness. As such, the value 24.67% illustrates the cost of equity-awarded models focused on risk or poverty. For example, the equity-only model covers 49.47% (objective 2) of the population, while the risk-focused model covers just 11.25% (objective 1), underscoring the inherent trade-offs in policy-driven allocation strategies. In contrast, the average coverage across all locations under this population-based baseline is just 8.85%, substantially lower than the 24.67% achieved by the bi-objective model. This disparity illustrates how prioritizing population size alone fails to capture broader equity and risk considerations, leaving vulnerable, low-population, high-risk areas without access to critical services. This comparison underscores the value of incorporating multidimensional criteria beyond a simple population size into service allocation decisions. However, even the relatively improved coverage achieved by the bi-objective model is constrained by systemic limitations in available resources and facility capacity.

This limited average coverage highlights the structural limitations embedded in the current resource allocation strategy. The main restrictions affecting coverage are related to the capacity of the facility and the overall resource budget available for the opening and operation of centers. For example, with only two centers allowed under the current budget, many rural or remote municipalities remain underserved. Relaxing budget constraints, allowing for a third center, or increasing hourly capacity could significantly improve geographic coverage and accessibility. Using a basic arithmetic approach, we can estimate potential gains in different scenarios. For instance, if a third center were added in strategically underserved locations and assuming it could replicate the average local coverage achieved by each of the two existing centers (about 12.33% each), overall average coverage could increase from 24.67% to approximately 37%. Similarly, increasing the service capacity of existing centers by 50% could proportionally increase the coverage by about half of the current value (an additional 12.3%), resulting in 37% average coverage. A combined strategy that adds one center and increases capacity by 25% would reasonably lead to average coverage values close to or exceeding 40%. These approximations illustrate that even modest budget increases can yield significant improvements in service reach across underserved locations. These hypothetical improvements highlight the importance of resource planning decisions in expanding service reach. To better understand how each modeling approach translates budget constraints into service delivery, we examine the estimated treatment capacities between selected facilities under each objective function.

**Table 6:** Percentage of demand covered ($<40$ km)

	Bi-objective		Objective 1		Objective 2		Baseline	
	Prevention	Mitigation	Prevention	Mitigation	Prevention	Mitigation	Prevention	Mitigation
	Avg: 14.05	Avg: 35.29	(Avg: 12.18)	(Avg: 10.31)	(Avg: 39.43)	(Avg: 49.46)	(Avg: 8.8)	(Avg: 8.9)
Barranquilla	3.60	19.72	8.18	34.07	0.24	0.17	8.17	34.06
Baranoa	4.7	3.27	4.7	3.27	4.7	100	4.69	3.27
Campo de La Cruz	12.81	8.91	12.81	8.91	12.81	8.91	0.00	0.00
Candelaria	18.33	100	18.33	12.76	18.33	100	0.00	0.00
Galapa	4.83	3.36	4.83	3.36	4.83	3.36	4.82	3.36
Juan de Acosta	13.95	9.72	13.95	9.72	100	100	0.00	0.00
Luruaco	10.4	100	10.4	7.24	100	100	0.00	0.00
Malambo	2.29	1.59	2.29	1.59	2.29	1.59	2.28	1.59
Manatí	14.79	10.3	14.79	10.3	14.79	10.3	0.00	0.00
Palmar de Varela	10.13	7.05	10.13	7.05	10.13	7.05	10.12	7.05
Piojó	44.34	100	44.34	30.86	100	100	0.00	0.00
Polonuevo	16.1	11.21	16.1	11.21	16.1	100	16.10	11.21
Ponedera	12.29	100	12.29	8.55	100	100	0.00	0.00
Puerto Colombia	5.95	4.14	5.95	4.14	5.95	4.14	5.94	4.14
Repelón	11.18	7.79	11.18	7.79	11.18	100	0.00	0.00
Sabanagrande	9.06	6.31	9.06	6.31	9.06	6.31	9.06	6.30
Sabanalarga	62.40	100	3.14	2.19	17.47	100	0.00	0.00
Santa Lucía	18.28	12.72	18.28	12.72	18.28	12.72	0.00	0.00
Santo Tomás	9.82	6.84	9.82	6.84	9.82	6.84	9.82	6.83
Soledad	0.49	0.34	0.49	0.34	0.49	0.34	0.48	0.33
Suan	24.54	17.06	24.54	17.06	24.54	17.06	0.00	0.00
Tubará	16.86	17.06	16.86	11.74	100	100	16.86	11.73
Usiacurí	23.92	16.64	23.92	16.64	23.92	16.64	0.00	0.00

Table 7 presents the estimated total capacity of the health center to be opened for each proposed model. Considering objective function 1, the Barranquilla health center would be able to accommodate 23,744 therapies, with 3,487 units designated for prevention and 20,257 for mitigation purposes. Similarly, the Baranoa health center would provide 4,160 therapies, evenly distributed between prevention and mitigation (2,080 each). Applying objective function 2, the Luruaco health center would handle 8,889 therapies, 2,235 allocated for prevention, and 6,654 for mitigation. In contrast, the Sabanalarga health center would accommodate 13,320 therapies, with 3,332 intended for prevention and 9,988 for mitigation. With the implementation of the proposed bi-objective model, the health center in Barranquilla would administer 15,089 therapies, dividing them into 2,080 for prevention and 13,009 for mitigation. In addition, the Sabanalarga health center would provide 12,815 therapies, with 3,487 allocated for prevention and 9,328 for mitigation. In particular, this allocation ensures an equitable distribution of services between different demand zones. It is essential to mention that Barranquilla would account for 54% of the treatments, while Sabanalarga would cover only 46% of the total treatments available in a year within budget constraints. In summary, under objective function 1, Barranquilla offers the highest capacity, followed by Baranoa. In contrast, Objective function 2 prioritizes rural areas, with Sabanalarga having the highest capacity, followed by Luruaco.

**Table 7:** Estimated total hourly capacity of the health center to be opened

	Bi-objective		Objective 1		Objective 2	
	Prevention	Mitigation	Prevention	Mitigation	Prevention	Mitigation
Barranquilla	2080	13009	3487	20257	-	-
Baranoa	-	-	2080	2080	-	-
Campo de La Cruz	-	-	-	-	-	-
Candelaria	-	-	-	-	-	-
Galapa	-	-	-	-	-	-
Juan de Acosta	-	-	-	-	2235	6654
Luruaco	-	-	-	-	-	-
Malambo	-	-	-	-	-	-
Manatí	-	-	-	-	-	-
Palmar de Varela	-	-	-	-	-	-
Piojó	-	-	-	-	-	-
Polonuevo	-	-	-	-	-	-
Ponedera	-	-	-	-	-	-
Puerto Colombia	-	-	-	-	-	-
Repelón	-	-	-	-	-	-
Sabanagrande	-	-	-	-	-	-
Sabanalarga	3487	9328	-	-	3332	9988
Santa Lucía	-	-	-	-	-	-
Santo Tomás	-	-	-	-	-	-
Soledad	-	-	-	-	-	-
Suan	-	-	-	-	-	-
Tubará	-	-	-	-	-	-
Usiacurí	-	-	-	-	-	-

Taken together, Tables 4, 5, 6, and 7 provide a view of how the proposed optimization model allocates resources between locations, balancing coverage levels, service hours, and total facility capacity under different objective functions. These results demonstrate not only the operational trade-offs between risk reduction and equity but also the spatial implications of each allocation strategy. Beyond this quantitative analysis, it is equally important to reflect on the quality and representativeness of the indicators driving the model, particularly those derived from social media data. While part of the allocation, especially in Tables 6 and 7, is influenced by the risk index that incorporates sentiment signals from X (formerly Twitter), this component introduces inherent uncertainties. Although X offers a rich source of public discourse related to psychoactive substance use, it does not necessarily reflect the full diversity of the at-risk population. Vulnerable groups, including individuals from rural areas, low-income backgrounds, or those without consistent internet access, are often underrepresented on such platforms. As a result, the sentiment and thematic data extracted from X can disproportionately capture the perspectives of more connected populations, potentially overlooking marginalized locations that face the most significant barriers to healthcare. To mitigate this limitation, the sentiment-based risk component ($\psi _i$) was intentionally weighted as only one-third of the composite risk index. However, its inclusion has a measurable influence on the outcomes of the model and must be interpreted with caution. Therefore, we insist that this variable serves as a complementary proxy, valuable to capture dynamic public sentiment but insufficient as a standalone indicator of risk. Future research should aim to integrate social media data with more representative sources, such as national health surveys, administrative datasets, other social networks, or field-level assessments, to ensure a more inclusive and robust basis for resource allocation in drug prevention and mitigation strategies.

### Sensitivity analysis

**Fig. 5:**
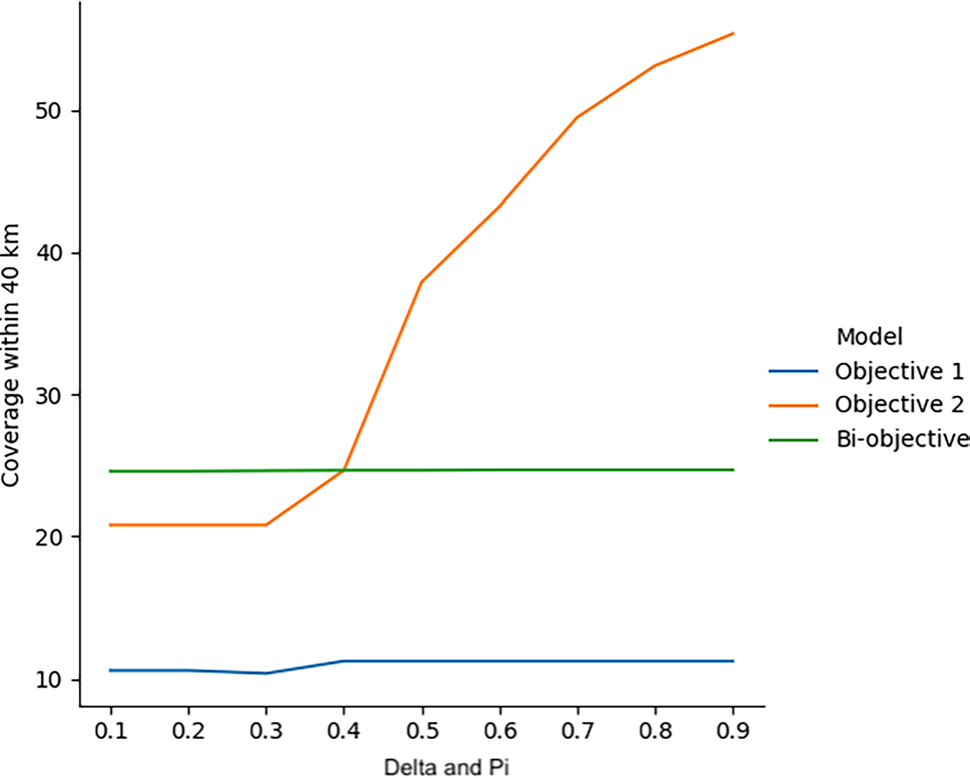
Sensitivity analysis of delta ($\delta$)

In addition to analyzing total capacity distribution, we conducted a sensitivity analysis to evaluate the stability of the bi-objective model under varying parameter configurations. The objective functions incorporate a linear combination of distance, risk, and equity, controlled by the weighting parameters such as $\delta$ and $\pi$. For clarity, these parameters were used directly in the formulation of the objective functions: $\delta$ in Objective Function 1 and $\pi$ in Objective Function 2. In the case of the bi-objective model, both parameters are applied simultaneously. Accordingly, the x-axis in Fig. 5 represents $\delta$ when referring to Objective Function 1, $\pi$ for Objective Function 2, and both when analyzing the bi-objective model. To assess robustness, we varied $\delta$ and $\pi$ from 0.1 to 0.9, observing its impact on the percentage of coverage within 40 km. The results show that the bi-objective model achieves consistent, moderate coverage ($\approx 24.6\%$) across all $\delta$ and $\pi$ values, indicating robustness regardless of how priorities shift between distance and risk-equity trade-offs. In particular, as $\delta$ and $\pi$ increases (greater emphasis on risk and equity), coverage achieved by Objective 2 improves significantly, surpassing both Objective 1 and the bi-objective model beyond $\pi = 0.4$. This highlights that while the bi-objective model provides stable, balanced solutions, models prioritizing risk and equity may outperform it in specific metrics like coverage when those factors dominate the decision criteria.

**Fig. 6:**
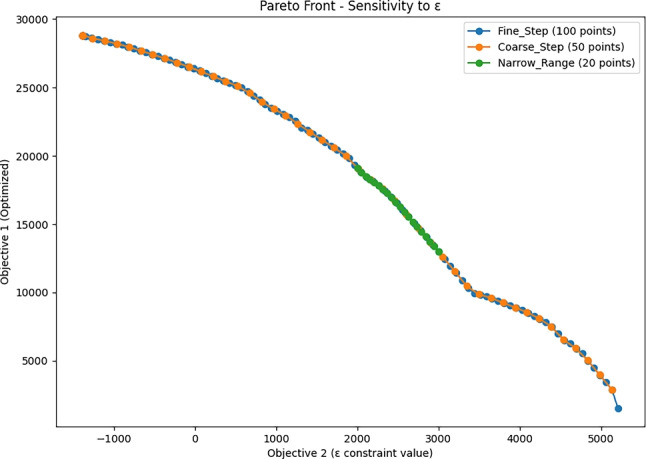
Sensitivity analysis of $\epsilon$

Furthermore, recognizing the sensitivity of the $\epsilon$-constraint method to the selection of $\epsilon$ values, we performed a systematic sensitivity analysis by varying $\epsilon$ in three distinct configurations: (i) fine step increments of 100 points across the entire feasible range, (ii) coarse step increments of 50 points, and (iii) a focused analysis with 20 points within a critical trade-off interval ($\epsilon \in [2000, 3000]$). The resulting Pareto frontiers (Fig. 6) show a continuous and expected trade-off behavior between the objectives. As anticipated, regions of the frontier corresponding to sharp trade-offs displayed greater sensitivity to selection $\epsilon$. Specifically, the maximum relative variation in Objective 1 between consecutive $\epsilon$ points was 45.66% for the fine configuration, 31.74% for the coarse configuration, and only 1.11% within the narrow critical range. The significantly lower variation within the focused interval confirms the numerical stability of the model in the most operationally relevant portion of the frontier, where decision-makers typically prioritize solutions. These findings validate both the robustness and the expected sensitivity patterns of the $\epsilon$-constraint method when applied to resource allocation under competing objectives.

In summary, the sensitivity analyses confirm that the proposed bi-objective model and solution approach are robust under varying parameter configurations, providing decision makers with reliable and adaptable resource allocation strategies. Building on this, we complement the quantitative modeling with an analysis of public discourse surrounding drug consumption, offering additional information to inform context-sensitive interventions.

## Complementary Analysis: X Topic Modeling

Social networks like Facebook, LinkedIn, and X have become indispensable sources of information for a wide range of users [70–73]. In this study, we built an algorithm to extract posts from X between 2019 and 2020 related to psychoactive substances, storing them in a PostgreSQL database. These posts were sourced from areas with high drug consumption ($\alpha _i$), to perform a complementary topic modeling analysis.

This analysis was independent of the sentiment classification process used to estimate the risk index ($\psi _i$) and instead served a separate purpose: to explore the prevalent themes and concerns surrounding drug use based on public discourse. This complements the optimization model by offering contextual understanding for potential interventions identified through the location-allocation framework. To maintain focus on the core modeling approach, the full methodology for data extraction, processing, and topic modeling, as well as detailed results, are presented in Appendix C.

## Conclusion

This research demonstrated an integrated framework to improve the effectiveness and equity of drug abuse prevention and treatment by combining a location-allocation bi-objective optimization model with social media sentiment analysis. The model optimized the location of health centers, counseling services, and harm reduction programs in areas with high drug-related risks, with the goal of maximizing accessibility while promoting social equity under resource constraints.

We evaluated the performance of the model using demand coverage, equitable distribution of resources, and reduction in patient travel distances. The proposed approach achieved an average coverage of 24.67% within a 40 km radius, nearly tripling the 8.85% coverage obtained by a baseline population-based heuristic that concentrated services only in major urban centers. The model also ensured service availability in both high-risk urban areas and underserved rural locations, illustrating its ability to balance efficiency and equity objectives. The computational experiments, implemented in Python and solved using Gurobi Optimizer v9.5.2, consistently reached near-optimal solutions with an optimality gap below $10^{-4}$. Sensitivity analyzes confirmed the robustness of the bi-objective model across varying parameter configurations, including adjustments to risk-equity weights ($\alpha$) and $\epsilon$-constraint settings. In particular, expanding the number of health centers from two to three or increasing service capacity by 50%, could increase the average coverage to approximately 37%, highlighting the significant impact of modest budget adjustments.

In parallel, a complementary topic modeling analysis using Latent Dirichlet Allocation (LDA) extracted key themes from more than 4,000 social media posts related to the use of psychoactive substances. The results revealed public concerns about drug trafficking, consumption patterns, legalization debates, and the societal impacts of addiction. These findings reflected community-level concerns and social narratives surrounding drug use, providing valuable qualitative context to support intervention design. Although topic modeling did not directly influence the risk index used for optimization, it provided valuable qualitative insights to inform outreach strategies and improve the design of the intervention.

In general, this study contributed to the literature in several ways: (i) it integrated social media-derived risk signals into an optimization framework for healthcare resource planning; (ii) it demonstrated the potential of bi-objective models to balance trade-offs between risk reduction and equity; and (iii) it provided policymakers with actionable recommendations to guide resource allocation and infrastructure investment.

From a policy perspective, the findings underscored the need for data-informed phased implementation strategies, prioritizing areas with the highest combined risk and socioeconomic vulnerability. Although urban centers maximize short-term demand coverage, expanding access to rural underserved communities promotes long-term reductions in health disparities. Furthermore, the ability of the model to visualize trade-offs offers decision makers the flexibility to align resource deployment with regional priorities and stakeholder preferences. In conclusion, this research delivered more than an optimized allocation scheme; it provided a transparent and adaptable platform to inform health policy, improve intervention effectiveness, and improve equitable access to drug prevention and treatment services. By integrating quantitative optimization with qualitative social insights, the proposed approach contributes to building more resilient, inclusive, and responsive healthcare systems.

### Limitations and future work

Future research should aim to develop more advanced location-allocation models that incorporate uncertainty and stochastic variation in both drug consumption patterns and resource availability. Furthermore, enhancing the sentiment analysis component (${\psi _i}$) requires further attention, particularly through sensitivity analyses that examine the robustness of the optimization results under different assumptions about social media bias. Researchers should also continue to improve the quality and representativeness of indicators based on social media, especially addressing geolocation limitations and demographic bias.

Although X (formerly Twitter) offers access to large-scale real-time social discourse, its user base may not accurately reflect the demographics of those most at risk for substance use or those facing the greatest barriers to healthcare. Vulnerable populations, such as individuals in rural areas or those with limited digital access, can be underrepresented, potentially introducing demographic and behavioral sampling bias in ${\psi _i}$. This limitation reinforces the need to triangulate sentiment-based indicators with other data sources. In this sense, future work should focus on improving the representativeness and reliability of sentiment-derived indicators. This may involve integrating slang-inclusive lexicons, improving geolocation precision, or combining social media data with alternative risk metrics such as community-level health surveys or administrative data. In addition, future studies could consider whether metadata typically excluded during preprocessing, such as the number of likes, retweets, or replies, may provide useful signals for refining sentiment estimation. While these elements are often treated as irrelevant for textual analysis, their inclusion could enhance the interpretability of the intensity of public sentiment and engagement around substance use topics.

Finally, special attention should be paid to how the absence of social media data affects risk estimation. In cases where no social media posts are available for a given location, the social media risk component (${\psi _i}$) is assigned a value of zero. However, this does not imply that the location is risk-free. The risk index ($R_i^k$) still incorporates the demand for psychoactive substances (${\alpha _i}$) and contextual risk factors (${\gamma _i}$), both of which remain valid inputs regardless of the availability of social media. However, this modeling assumption introduces potential underestimation in areas with limited online activity, particularly in rural or underserved communities.

## Supplementary Information

Below is the link to the electronic supplementary material.

Supplementary file 1 (pdf 155 KB)

Supplementary file 2 (pdf 97 KB)

Supplementary file 3 (pdf 660 KB)

## Data Availability

The authors confirm that the data supporting the findings of this study are available within the article.
