# Supplementary material for: Bi-objective location-allocation model of interventions in high drug consumption areas incorporating X topic modeling
Source: Health Care Manag Sci. 2026 May 8;29(2):20. doi: 10.1007/s10729-025-09753-3 (PMC13156152; doi:10.1007/s10729-025-09753-3)
Supplement: Supplementary file 1 — (pdf 155 KB) [file 10729_2025_9753_MOESM1_ESM.pdf]

Appendix A. Algorithms

August 1, 2025

1 Algorithm A1. Epsilon Constraint Method

Given a multi-objective optimization problem with n decision variables and m objectives (minimize or maximize). Let $f_1(x)$ be an objective function subject to:

$$g_1(x) \leq 0, ..., g_k(x) \leq 0, ..., g_m(x) \leq \epsilon_m \quad (1)$$

where x is the vector of decision variables, $f_1(x)$ is the primary objective function to optimize, $g_1(x), \ldots, g_k(x)$ are the constraints that define the feasible region of the problem and ϵ_m is a positive scalar that determines the maximum value of the $m-th$ objective. The epsilon constraint method transforms the multi-objective optimization problem into a set of single-objective optimization problems, each with a different value of epsilon. By solving each of these problems, the method produces a set of Pareto-optimal solutions, which represent the trade-off between the objectives. Once the set of Pareto-optimal solutions has been determined, the values with the smallest Euclidean distance between the ideal point (the objective value obtained without applying the epsilon constraint) and the Pareto frontier are selected. The mathematical procedure of the epsilon constraint method can be summarized in the algorithm 1. Examples using multi-objective optimization approaches to improve resource utilization in health systems can be found in [1, 2, 3].

Algorithm 1 Epsilon constraint method

```
Choose one objective as the primary objective f_1
Determine x^1 an optimal solution for f_1
A ← {x^1}
ε_2 ← f_2(x^1) + δ
while max_{x∈X}{f_1(x)‖f_2(x) ≥ ε_2}isfeasible do
    x̂ ← max_{x∈X}{f_1(x)‖f_2(x) ≥ ε_2
    A ← A ∪ x̂
    ε_2 ← f_2(x̂) + δ
end while
Filter dominated solutions in A
```

2 Algorithm A2. Latent Dirichlet Allocation (LDA)

Algorithm 2 Latent Dirichlet Allocation

Let $W_{d,j}$ be the frequency of term j in document d
Let z be the topic assignments and counts $n_{d,l}, n_{l,w}, n_l$
Randomly initialize z and increment counters
for $i = 0 \to N - 1$ **do**
  word $\leftarrow w[i]$
  topic $\leftarrow z[i]$
  $n_{d,topic} -= 1; n_{word,topic} -= 1; n_{topic} -= 1$
  for $l = 0 \leftarrow L - 1$ **do**
    $p(z = l \| \cdot) = (n_{d,l} + \theta_l) \frac{n_{l,w} + \beta_w}{n_l + \beta \times W_{d,j}}$
  end for
  topic $\leftarrow$ sample from $p(z \| \cdot)$
  $z[i] \leftarrow$ topic
  $n_{k,topic} += 1; n_{word,topic} += 1; n_{topic} += 1$
end for
Return topic assignments z and $n_{d,l}, n_{l,w}, n_l$

References

[1] Arriz-Jorquiera, M., Acuna, J. A., Rodríguez-Carbó, M., Zayas-Castro, J. L. (2024). Hospital food management: a multi-objective approach to reduce waste and costs. Waste Management. 175, 12–21. DOI: 10.1016/J.WASMAN.2023.12.010.

[2] Acuna, J. A., Zayas-Castro, J. L., Charkhgard, H. (2020). Ambulance allocation optimization model for the overcrowding problem in US emergency departments: A case study in Florida. Socio-Economic Planning Sciences. 71, 100747. DOI: 10.1016/J.SEPS.2019.100747.

[3] Acuna, J. A., Zayas-Castro, J. L., Feijoo, F., Sankaranarayanan, S., Martinez, R., Martinez, D. A. (2022). The Waiting Game – How Cooperation Between Public and Private Hospitals Can Help Reduce Waiting Lists. Health Care Management Science, 2022, 25(1), 100–125. DOI: 10.1007/S10729-021-09577-X/TABLES/4.
