# Supplementary material for: Bi-objective location-allocation model of interventions in high drug consumption areas incorporating X topic modeling
Source: Health Care Manag Sci. 2026 May 8;29(2):20. doi: 10.1007/s10729-025-09753-3 (PMC13156152; doi:10.1007/s10729-025-09753-3)
Supplement: Supplementary file 2 — (pdf 97 KB) [file 10729_2025_9753_MOESM2_ESM.pdf]

Appendix B

August 1, 2025

1 SQL Query for Data Collection

This appendix describes the data extraction method from X (formerly Twitter) used in the topic modeling analysis. The data collection involved the use of the Tweepy package and the academic access endpoint of the X API. The detailed procedure involved the following steps:

- Step 1 (Data Collection): Collect X data using the X API importing the Tweepy package. Then, we used the URL `https://api.twitter.com/2/tweets/search/all` under the research academic access to extract posts based on the following query:

  ```
  query_params = {'query':'( droga OR drogadicto OR
      opioide OR opioides OR opiaceo OR opiaceos OR
      morfina OR oxicodona OR oxicotin OR fentanilo OR
      durogesic OR hidromorfona OR meperidina OR
      tramadol OR tramal OR hidrocodona OR vicodin OR
      sinalgen OR heroina)'}
  ```

  Then, the posts are stored in a database in Postgres SQL.

- Step 2 (Data Cleaning): Pre-process the collected posts by removing URLs, special characters, stop words, and irrelevant content such as reposts or replies. In addition, tokenization, stemming, and lemmatization are performed to standardize the text.

- Step 3 (Feature Extraction): Convert the pre-processed posts into a numerical representation using a vectorization technique such as TF-IDF, TF, or bag-of-words. This step involves creating a term document matrix where each row represents a post and each column represents a unique term or word.

- Step 4 (Topic Modeling): Apply a clustering or decomposition algorithm to the term-document matrix to identify the underlying topics in the posts. In this research, we used Latent Dirichlet Allocation (LDA), a probabilistic topic modeling algorithm, to identify the underlying topics in a collection of documents [1]. The mathematical procedure for LDA can be described in algorithm A2 (see Appendix A).

- Step 5 (Topic Evaluation): Evaluate the quality and coherence of the identified topics using metrics such as perplexity and coherence. A higher coherence score indicates that the topic is more meaningful and interpretable, while a lower score implies randomness or lack of coherence [2]. On the other hand, perplexity is a commonly used measure to evaluate the performance of language models, including topic models. It assesses how accurately a language model can predict a waiting set of documents, and a lower score indicates higher precision [3].
- Step 6 (Interpretation): Interpret the identified topics by examining the top terms and their co-occurrence patterns.

Note: As is common with social media data, posts were often informal, featuring creative grammar, slang, emoticons, and abbreviations, making topic extraction more challenging than standard text sources [4]. To ensure data quality, we applied the framework presented in [5]. A post is chosen if it contains two or more terms from the ontology. This scheme seeks through each user's post in the corpus to make sure that it contains related terms from the ontology. We used the ontology proposed by [5].

References

[1] Blei, D. M., Ng, A. Y., Jordan, M. I. (2003). Latent Dirichlet Allocation. Journal of Machine Learning Research, 3:993-1022.

[2] Mimno, D., Wallach, H. M., Talley, E., Leenders, M., McCallum, A. (2011). Optimizing Semantic Coherence in Topic Models. Proceedings of the 2011 Conference on Empirical Methods in Natural Language Processing, 262-272.

[3] Jelinek, F. (1980). Interpolated estimation of Markov source parameters from sparse data.

[4] Stieglitz, S., Mirbabaie, M., Ross, B., (2018). Social media analytics – Challenges in topic discovery, data collection, and data preparation. International Journal of Information Management, 39:156-168. DOI: `10.1016/J.IJINFOMGT.2017.12.002`.

[5] Nasralah, T., El-Gayar, O., Wang, Y. (2020). Social media text mining framework for drug abuse: Development and validation study with an opioid crisis case analysis. Journal of Medical Internet Research. 22(8):e18350. DOI: `10.2196/18350`.
