# Supplementary material for: Bi-objective location-allocation model of interventions in high drug consumption areas incorporating X topic modeling
Source: Health Care Manag Sci. 2026 May 8;29(2):20. doi: 10.1007/s10729-025-09753-3 (PMC13156152; doi:10.1007/s10729-025-09753-3)
Supplement: Supplementary file 3 — (pdf 660 KB) [file 10729_2025_9753_MOESM3_ESM.pdf]

Appendix C

August 1, 2025

1 Extended Topic Modeling Analysis

The topic modeling section of the manuscript, now summarized in Section 3, has been extended here to readers interested in the full results of the Latent Dirichlet Allocation (LDA) modeling process. Although the core of this work is the bi-objective optimization model, topic modeling offers valuable insights into the societal discourse surrounding drug use, supporting contextual understanding of potential interventions. The final model revealed nine themes, ranging from drug cartel activities and legalization debates to societal impacts and personal responsibility.

The detailed procedure involved the following steps (see Appendix B for details on the SQL query used for data collection):

- Data collection: 4,363 posts from the Department of Atlántico (Colombia) were collected using the X API and stored in PostgreSQL.
- Data cleaning: 1,274 posts were removed based on quality criteria; the remaining 3,089 posts were retained.
- Feature extraction: The texts were vectorized using standard techniques like TF-IDF. Figure 1 presents an example of the posts collected
- Topic modeling: LDA was applied. The optimal number of topics was determined to be 9, based on coherence and perplexity scores (perplexity: -40,814).
- Topic Evaluation: Figure 2 illustrates the performance of topic coherence in varying topic counts.
- Interpretation: The topics were interpreted through word clouds (Figure 3) and descriptive summaries.

1.1 Results Summary:

- T1 (Drug cartel activities and global impacts): Colombian former president Duque, was likely to be involved in efforts to combat drug cartels,

which are known to produce and distribute illegal drugs [1]. “Fumar”, or smoking, can also be linked to this issue, as tobacco is a legal drug that can have harmful effects on health, and some cartels may also be involved in the illegal tobacco trade. The impact of drug cartels can extend beyond Colombia to the rest of the world, as they can contribute to issues such as drug addiction, crime, and political instability. Overall, these words can be connected to the theme of drug-related issues and their global impact [2, 3].

- T2 (Drug use in public spaces and across countries): “Droga”, or drug, are substances that can have negative effects on health and well-being. “Meter”, or to use, can be linked to drug use, as it refers to the act of taking drugs. “Parque”, or park, may represent a public space where drug use can occur, highlighting the issue of drug use in public areas. “Países”, or countries, suggests that this issue can have different manifestations and consequences in different parts of the world. Finally, “decisión”, or decision, could represent the need for policymakers and individuals to make choices regarding drug use and its impact on society. Overall, these words can be connected to the theme of drug use and its impact on public spaces, individual health, and society as a whole, emphasizing the importance of making informed decisions and taking appropriate actions to address this issue [4, 5, 6].

- T3 (Cannabis use and personal responsibility): Cannabis is a plant that can be used for recreational or medicinal purposes, and its consumption (“consumo”) can have both positive and negative effects on the health and well-being of individuals. The term “persona”, or person, emphasizes the importance of considering the individual and their unique circumstances when it comes to cannabis consumption. Finally, “produce”, or to produce, can refer to the cultivation and production of cannabis, highlighting the need for responsible and sustainable practices in this industry. Overall, these words can be connected to the theme of cannabis production and consumption and its impact on individuals and society, emphasizing the need for informed decision-making and responsible practices to ensure the safety and well-being of all stakeholders involved [7, 8].

- T4 (Legal and ethical considerations surrounding drug use): “Ser”, or to be, can refer to the innate characteristics and values that individuals possess, highlighting the importance of personal responsibility in making decisions related to drug use. “Hacer”, or to do, can refer to the actions and behaviors individuals take regarding drugs, such as being responsible in the use and distribution of drugs. “Gente”, or people, represents the wider community in which individuals live and interact, emphasizing the importance of responsible and ethical behavior towards others, particularly in the context of drug use. “Favor”, or favor, can represent the act of helping others, emphasizing the importance of supporting those who may be struggling with drug addiction or abuse. Legal, or legal, highlights

the importance of following laws and regulations related to drug use and drug-related activities, emphasizing the importance of preventing harm and promoting public safety. Finally, the word drug itself emphasizes the importance of responsible and ethical decision-making when it comes to drug use, as the consequences of drug use can have significant impacts on both individuals and society as a whole. Overall, these words can be connected to the theme of responsible and ethical behavior in the context of drug use and drug-related activities, emphasizing the importance of personal responsibility, empathy, and public safety [9, 10].

- T5 (The debate over marijuana legalization): "Legalización", or legalization, refers to the process of making marijuana legal for consumption, sale, and distribution. "Millones", or millions, refers to the large number of people who may be affected by the legalization of marijuana, both positively and negatively. "Vida", or life, highlights the potential impact of marijuana use on individuals' health and well-being, both physically and mentally. "Hierba", or herb, refers to marijuana as a natural plant-based substance, which may be viewed positively or negatively depending on cultural and societal attitudes toward drug use. "Droga", or drug, is a more general term that can be used to refer to any substance that has the potential to be abused or cause harm. "Quiere", or wants, suggests the desire or interest that some individuals or groups may have in legalizing marijuana. Consume, or consume, refers to the act of using marijuana, emphasizing the potential impact of legalization on individuals' behavior and consumption patterns. Overall, these words can be connected to the ongoing debate and controversy surrounding the legalization of marijuana and the impact it may have on individuals and society, including issues related to health, legality, and cultural attitudes toward drug use [11, 12].

- T6 (Drug addiction and its impact on vulnerable populations): "Adicción", or addiction, refers to the compulsive and harmful use of drugs that can lead to physical and mental health issues. "Amigo", or friend, may highlight the role that social networks and peer pressure can play in drug use and addiction. "Droga", or drug, refers to the substances that can lead to addiction and other harmful effects. Cartel and "FARC-Santos" refer to criminal organizations that are involved in drug trafficking and may exacerbate issues related to drug addiction and violence. "Menores", or minors, refers to young people who may be particularly vulnerable to the negative effects of drug use and addiction. Together, these words suggest the complex and interconnected nature of the issue of drug addiction, which involves individual behavior, social networks, criminal organizations, and societal factors such as access to healthcare and education. They also highlight the need for comprehensive and evidence-based approaches to address the issue of drug addiction and its impact on individuals and society [13, 14].

- T7 (Violence, corruption, and organized crime): "Droga", or drug, refers

to the substances that can lead to addiction and violence, and the devastating consequences of drug-related deaths. “Muerte”, or death, highlights the tragic toll of drug-related violence on individuals and communities, including innocent bystanders and those caught in the crossfire. “Corrupción”, or corruption, emphasizes the corrosive impact of the illicit drug trade and the criminal enterprises it fuels on governance, law enforcement, and society at large. “Poder”, or power, highlights the ways in which drug-related violence and corruption are perpetuated by those who hold political, economic, or social power, and the challenges of addressing these entrenched interests. “Grande”, or big, underscores the scale and complexity of the drug trade and its impact on society, including the significant resources required to address it. “Problema”, or problem, underscores the urgency of addressing the root causes of drug-related violence and corruption, including poverty, inequality, and social exclusion. “Corrupción” and “Partido” both suggest the need for comprehensive and coordinated efforts to combat corruption and address the political and institutional factors that allow it to thrive. Together, these words highlight the need for a multi-faceted approach to address the complex and interconnected issues of drug-related violence and corruption, including a focus on prevention, law enforcement, and systemic reform [15].

- T8 (Alcohol, sex, and addiction in urban settings): “Alcohol” represents the consumption of alcohol, which can lead to addiction and negative health consequences. “Sexo” refers to sexual activity, which can also have negative consequences when not practiced safely and consensually. “Barrio”, or neighborhood, suggests that these issues may be particularly acute in urban areas with high levels of poverty, crime, and social inequality. “Video” may represent the role of media and technology in promoting and normalizing these behaviors, as well as potential negative consequences such as addiction to video games or pornography. “Vicio”, or addiction, highlights the risk of developing problematic and potentially harmful behaviors associated with alcohol, sex, or other addictive substances or activities. Together, these words suggest the need for education, awareness, and support services to address the negative consequences of these behaviors and promote healthier, safer lifestyles in urban communities [16, 17].

- T9 (Societal impacts of drug-related crime and overdose):

  This topic includes terms such as “droga”, “muerte”, “social”, “dosis”, “guerra”, “violadores”, “homosexuales”, “ladrones”, and “secuestradores”, which reflect a wide spectrum of public discourse on the broader consequences of drug use. The presence of terms like “droga”, “dosis”, and “muerte” highlight concerns about overdose and drug-related mortality. Words such as “guerra” and “social” suggest discussion of the societal battle against drug trafficking and its widespread impact on communities. The inclusion of “homosexuales” in Topic 9 requires careful interpretation. Rather than implying that homosexuality is a negative effect of drug use,

its presence likely arises from co-occurrence in social media narratives that highlight the heightened vulnerability of LGBTQ+ individuals to violence, mental health challenges, and substance use. According to the CDC's 2023 Youth Risk Behavior Surveillance System, LGBTQ+ youth report significantly higher rates of illicit drug use (15% vs. 8%) and suicide attempts (41% vs. 13%) compared to their heterosexual peers [18]. A 2022 PubMed meta-analysis also shows that transgender and gender-diverse youth are disproportionately affected by substance use and overdose, often linked to victimization and stigma [19]. These findings suggest the word "homosexuales" in T9 may reflect concern about social vulnerability rather than imply that homosexuality is inherently linked to crime. Additionally, some posts may carry stigmatizing language or prejudice, demonstrating how topic modeling can surface challenging or biased public discourse, reinforcing the need for contextual analysis of these emergent terms. Overall, these words highlight the complex and multifaceted nature of the drug problem, which requires a comprehensive approach that addresses not only the physical and psychological aspects of drug use but also the social, economic, and political factors that contribute to drug-related violence and insecurity [20].

1.2 Figures

:

X	El consumo intensivo de cannabis por parte de jóvenes con del estado de puede estar relacionado con un mayor riesgo de autolesión intentos de suicidio y muerte según estudios
X	134 desempleados esquineros y vagos fumadores de marihuana y que seguramente perdieron el ICFES no queda de otra
X	Falta que legalicen la marihuana ya logramos con el aborto

Figure 1: Sample of the collected posts

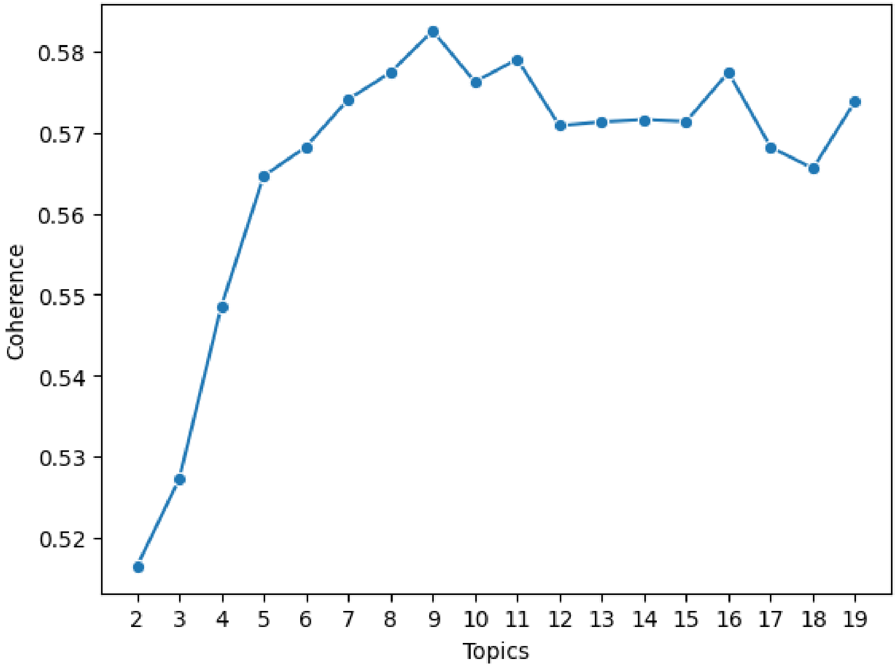

Figure 2: Coherence score over the number of topics

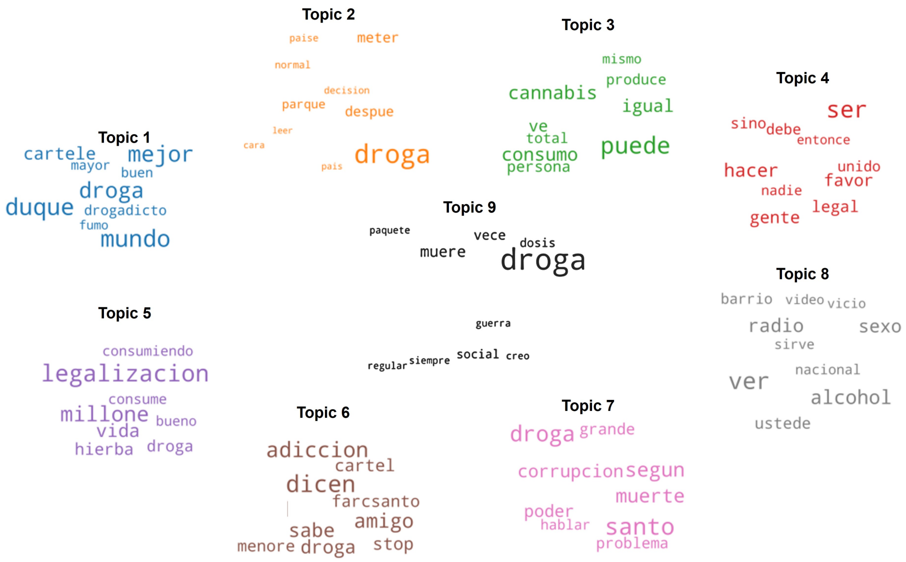

Figure 3: Top nine topic word clouds

1.3 Tables

Table 1: Topic Contribution

Topic Number	Topic Contribution	Keywords	Representative Text
1	0.4869	mundo, duque, mejor, droga, carteles, dio, drogadicto, buen, mayor, fumo	['salmo', 'impaciente', 'causa', 'malignos', 'envidia',]
2	0.5941	droga, meter, parque, normal, países, decisión, cara, país, leer	['hacen', 'lugares', 'rumbear', 'diferente', 'ambiente', 'meter', 'droga', 'diferente', 'ambiente']
3	0.5448	puede, cannabis, consumo, igual, ve, do, persona, produce, total, mismo	['total', 'igual', 'impunidad', 'droga', 'borrachera']
4	0.4676	ser, hacer, gente, favor, legal, debe, unido, nadie,	['droga', 'alterar', 'gene', 'químicas', 'diferencia', 'religión', 'rastafari', 'simple', 'planta', 'planta', 'respetar']
5	0.4898	legalización, millones, vida, dice, hierba, droga, quiere, consume, consumiendo, bueno	['invertido', 'miles', 'millones', 'pauta', 'promover', 'legalización', 'medio', 'twitter', 'llenos', 'testimonio', 'bueno', 'trabado']
6	0.5503	dicen, adicción, amigo, pue, sabe, droga, stop, cartel, farcsanto, menores	['outfit', 'natural', 'mugre', 'natural', 'toda', 'natural']
7	0.5765	santo, droga, muerte, corrupción, poder, grande, problema, hablar	['liberal', 'partido', 'corrupto', 'lopez_michelsen', 'gaviria', 'samper', 'santo', 'insistir', 'promueven', 'corrupción', 'promotores']
8	0.487	alcohol, radio, sexo, barrio, nacional, sirve, video, vicio	['vicio', 'sexo', 'gym', 'video', 'ver', 'dulce',]
9	0.516	droga, muere, vece, social, dosis, siempre, guerra, paquete, regular	['droga', 'violadore', 'homosexuales', 'ladrone', 'secuestradore', 'cómplice', 'narcoasesino', 'farcsa']

Note: The topic modeling approach illustrates the distribution of public posts in social media across various topics, predominantly highlighting the opioid crisis. The discussions range from the influence of drug cartels globally to the societal consequences of drug use, including personal responsibility in cannabis consumption and ethical behavior related to drug activities. Additionally, topics cover the debate over marijuana legalization, the extensive effects of drug addiction on society and vulnerable populations, and the pervasive violence and corruption linked to drug-related activities. This diverse spectrum of issues underscores the complexity of drug-related challenges, setting the stage for our proposed approach to addressing these concerns through strategic resource allocation and improved accessibility to prevention and treatment services.

Analyzing these topics gives us valuable insights into public sentiment, emerging trends, and key concerns surrounding drug use. This information could be crucial for tailoring prevention strategies, as it highlights areas of public interest and potential risk factors.

References

[1] Crisis group. (2021). Coca Eradication and Violence in Colombia. Available at: `https://www.crisisgroup.org/latin-america-caribbean/andes/colombia/87-deeply-rooted-coca-eradication-and-violence-colombia`.

[2] Merrill, S. (2008). Drugs and development: The global impact of drug use and trafficking on social and economic development. International Journal of Drug Policy. 19(6), 467-478. DOI: `10.1016/J.DRUGPO.2006.12.007`.

[3] Rubiano, A. M, Muñoz, J. H. M, Estebanez, G., Sanchez, A. I, Puyana, J. C.,, Puyana, J. C. (2018). Drugs, Violence and Trauma in the Colombian Context: A Health Care Point of View of a Human Rights Challenge,

Panamerican journal of trauma, critical care & emergency surgery, 7(2), 158. DOI: 10.5005/JP-JOURNALS-10030-1218.

[4] Geldart, R., Greene, C., Urbanik, M.-M., and Maier, K. (2024). They don't do nothing: Exploring marginalized people who use drugs experiences with and expectations of private security officers. SAGE Publications. Sage UK: London, England. DOI: https://doi.org/10.1177/17488958241249825.

[5] Piza, E. L., Wolff, K. T., Hatten, D. N., Barthuly, B. E. (2023). Drug overdoses, geographic trajectories, and the influence of built environment and neighborhood characteristics. Health & Place, 79, 102959. DOI: https://doi.org/10.1016/J.HEALTHPLACE.2022.102959.

[6] Hamzah, H., Burda, A., Jalal, A. (2023). Policy Analysis for Prevention of the Danger of Drugs Abuse for Young Generation. International Journal of Social Science and Human Research. 6(5), 123–134. DOI: https://doi.org/10.47191/IJSSHR/V6-I5-21.

[7] Florimbio, A. R., Walton, M. A., Duval, E. R., Bauermeister, J. A., Young, S. D., McAfee, J., Bonar, E. E. (2024). Direct and indirect effects of cannabis risk perceptions on cannabis use frequency. Addiction Research & Theory. 32(1), 68-73. DOI: https://www.tandfonline.com/doi/abs/10.1080/16066359.2023.2221029.

[8] MacDougall, C., Maston, M. (2023). Student perceptions of cannabis use. Journal of American College Health. 71(4), 1003-1017. DOI: https://www.tandfonline.com/doi/abs/10.1080/07448481.2021.1910272.

[9] Blancarte J. F., (2023). Conceptualizations of addiction in harm reduction strategies for effective and ethical UK drug policy. The University of Edinburgh. URL: https://era.ed.ac.uk/handle/1842/40686.

[10] Tersoo, A. D., Adeyongo, I. A. (2023). The Ethical Dimensions of the Use of Drugs and Substances among the Youth in Benue North-East Senatorial District. African Journal of Humanities and Contemporary Education Research, 13(1), 196-218. URL: https://publications.afropolitanjournals.com/index.php/ajhcer/article/view/661.

[11] Hinckley, J., Bhatia, D., Ellingson, J., Molinero, K., Hopfer, C. (2024). The impact of recreational cannabis legalization on youth: the Colorado experience. European Child and Adolescent Psychiatry, 33(3), 637-650. DOI: https://link.springer.com/article/10.1007/s00787-022-01981-0.

[12] Lévesque, G. (2023). Making sense of pot: conceptual tools for analyzing legal cannabis policy discourse. Critical Policy Studies, 17(1), 4-23. DOI: https://www.tandfonline.com/doi/abs/10.1080/19460171.2022.2044874.

[13] Charles, M. H. (2023). Strategic Offending: Colombia's Part-Time Child Drug Traffickers and Their Community, City and Country Lines. Youth Justice, 23(12). DOI: https://acortar.link/FzzOzR.

[14] Feixa, C., Ross, W., Lavielle, L., Chévez, C., Márquez, F. (2023). Researching Youth Street Groups in the Americas: Gangs, pandillas, maras, bandas. Transgang. URL: http://doi.org/10.31009/transgang.2023.wp07.1.

[15] Croci, G. (2023). Effectiveness and corruption in the criminal justice system of Latin America: An overview. International Journal of Comparative and Applied Criminal Justice, 47(12), 154-168. DOI: https://www.tandfonline.com/doi/abs/10.1080/01924036.2023.2292032.

[16] Salas-Ortiz, A., Moreno-Serra, R., Kreif, N., Suhrcke, M., and Casas, G. (2024). The effect of conflict-related violence intensity and alcohol use on mental health: The case of Colombia. SSM - Population Health, 25, 101626. DOI: https://doi.org/10.1016/J.SSMPH.2024.101626.

[17] Palomino, K., Berdugo, C. R., and Vélez, J. I. (2023). Leading consumption patterns of psychoactive substances in Colombia: A deep neural network-based clustering-oriented embedding approach. PLOS ONE, vol. 18, no. 8, p. e0290098. [Online]. Available: https://journals.plos.org/plosone/article?id=10.1371/journal.pone.0290098

[18] CDC. (2024). Health disparities among LGBTQ youth. Adolescent and School Health. Available at: https://www.cdc.gov/healthy-youth/lgbtq-youth/health-disparities-among-lgbtq-youth.html?utm_source=chatgpt.com.

[19] Eisenberg, M. E., Gower, A. L., Watson, R. J., Rider, G. N., Thomas, D. S., and Russell, S. T. (2022). Substance use behaviors among LGBTQ+ youth of color: Identification of the populations bearing the greatest burden in three large samples. Journal of Adolescent Health, vol. 71, no. 3, pp. 317–323. Available at: https://pubmed.ncbi.nlm.nih.gov/35715349/.

[20] Lisboa, C., Stuardo, V., Folch, C. (2023). Sexualized drug use among gay men and other men who have sex with men in Latin America: A description of the phenomenon based on the results of LAMIS-2018. PLOS ONE, 18(10), e0287683. DOI: https://journals.plos.org/plosone/article?id=10.1371/journal.pone.0287683.
